# Generative Adversarial Learning of Protein Tertiary Structures

**DOI:** 10.3390/molecules26051209

**Published:** 2021-02-24

**Authors:** Taseef Rahman, Yuanqi Du, Liang Zhao, Amarda Shehu

**Affiliations:** 1 Department of Computer Science, George Mason University, Fairfax, VA 22030, USA; trahman2@gmu.edu (T.R.); ydu6@gmu.edu (Y.D.); 2 Department of Computer Science, Emory University, Atlanta, GA 30322, USA; liang.zhao@emory.edu; 3 Center for Advancing Human-Machine Partnerships, George Mason University, Fairfax, VA 22030, USA; 4 Department of Bioengineering, George Mason University, Fairfax, VA 22030, USA; 5 School of Systems Biology, George Mason University, Manassas, VA 20110, USA

**Keywords:** protein modeling, tertiary structure, generative adversarial learning, deep learning

## Abstract

Protein molecules are inherently dynamic and modulate their interactions with different molecular partners by accessing different tertiary structures under physiological conditions. Elucidating such structures remains challenging. Current momentum in deep learning and the powerful performance of generative adversarial networks (GANs) in complex domains, such as computer vision, inspires us to investigate GANs on their ability to generate physically-realistic protein tertiary structures. The analysis presented here shows that several GAN models fail to capture complex, distal structural patterns present in protein tertiary structures. The study additionally reveals that mechanisms touted as effective in stabilizing the training of a GAN model are not all effective, and that performance based on loss alone may be orthogonal to performance based on the quality of generated datasets. A novel contribution in this study is the demonstration that Wasserstein GAN strikes a good balance and manages to capture both local and distal patterns, thus presenting a first step towards more powerful deep generative models for exploring a possibly very diverse set of structures supporting diverse activities of a protein molecule in the cell.

## 1. Introduction

Molecular structure and function are tightly related to each-other. Nowhere is this more evident than in proteins, where the three-dimensional/tertiary structure is central to recognition of molecular partners in the cell [1]. Recent news from the “Critical Assessment of protein Structure Prediction” (CASP) competition suggest that DeepMind’s AlphaFold2 has “solved” what is known as protein structure prediction [2], a problem that has been a 50-year old grand challenge in computational structural biology. The performance of AlphaFold2 on the test set of target proteins in CASP14 certainly suggests that an incredible milestone has been achieved.

Until we have access to the AlphaFold2 model or are able to reproduce it with fidelity, we will not know the true extent to which we now have a tool that we did not have before, giving us the ability to determine in silico a high-quality model of a biologically-active, tertiary structure of a given protein. It would be a wonderful tool, indeed. Currently, many regions of the protein universe are inaccessible in the wet or dry laboratory. Analysis of 546,000 unique proteins deposited in Swiss-Prot reveals that 44–54% of the proteome in eukaryotes and viruses and about 14% of the proteome in archaea lack structural and/or functional characterization [3].

Yet, focusing on obtaining just one structure betrays our understanding that protein molecules are inherently dynamic, harnessing their ability to access different tertiary structures to modulate interactions with different partners and so propagate signal across different cellular pathways [4]. Much work in protein modeling has focused on obtaining a broader view of the structure space accessible to them under physiological conditions [5]. This is an exceptionally challenging task [6], and the majority of research harnesses previous knowledge, whether via restricted physical models or existing structure data, in order to focus/bias a search algorithm on the relevant region(s) of a structure space that is too vast otherwise [7,8,9].

Current momentum in deep learning and the performance of generative adversarial networks (GANs) in generating credible data across domains [10,11,12] inspires us in this paper to investigate and evaluate GANs on their ability to generate physically-realistic protein tertiary structures. Work in this direction has recently started. For instance, work in [13] employs a long short-term memory GAN and trains it on backbone angles extracted from a specific subset of short protein structures containing only alpha helices. The model’s output structures are found to be quite varied in quality. The researchers report that some protein-like structures are found among those generated, which encourages them to further investigate GANs. In [14,15], the researchers take on this task but differ in two critical ways. First, they expand the training set to include proteins of diverse structures. Second, they change the representation of a tertiary structure from backbone angles to distance matrices that record in every entry the distance between the main-chain carbon atoms of two amino acids. We also find GANs in recent literature that are devoted to the narrow problem of protein structure prediction as a prediction of contacts problem [16]; that is, given a sequence of amino acids in a protein molecule, which pairs of main-chain CA atoms are spatially proximate? Work in [17] extends this line of work and uses GANs to predict actual Euclidean distances between pairs of main-chain CA atoms.

None of the above works, with the exception of [14,15], address the holistic setting of GANs for generating tertiary structures [18]. We note that more progress has been made recently with Variational Autoencoders (VAEs), which provide a generative framework complementary to GANs. We point here two representative works in this area [19,20]. However, these works train a VAE on structures generated for a specific protein molecule, and these structures are obtained from computational platforms, such as MD simulations [19] or protein structure prediction platforms, such as Rosetta [20]. None of these works leverage known experimental structures in the PDB, which has been the trend in the nascent sub-area of GANs for protein structure modeling as a way of learning from the actual ground truth distribution rather than other computational frameworks.

However, the current literature on GANs for protein structure modeling has not explored well a detailed analysis into the quality of the generated data. For instance, the issue of stability in training GANs is already well-recognized [21]. No indication is given into which training mechanisms, if any, result in convergence of both the discriminator and generator, particularly when the goal is to learn complex objects with inherent structure in them.

Moreover, the quality of the generated data is not related in any meaningful detail. To this day, we do not understand well the power (or lack of) of GANs for generating physically-realistic tertiary structures. For instance, what kind of tertiary structures can a GAN generate? Do the generated structures look like structures of proteins? Do they contain a backbone that connects consecutive amino acids? Do they contain the hallmark secondary structure elements (alpha helices, beta sheets, and so on). Are they compact, as is typically expected from a folded protein, or are they extended? These are important, fundamental questions if we want to start a line of research on GANs as viable frameworks for important problems that expand our view of a protein molecule beyond a single structure.

In this paper, motivated by our view of proteins as dynamic molecules, we seek to answer these questions. Employing a distance matrix representation of a tertiary structure as in [14,15], which we detail later in Section 2, we train various GAN models over diverse tertiary structures of proteins deposited in the Protein Data Bank (PDB) [22]. Unlike existing works [14,15], we generalize our observations on various training datasets of tertiary structures of protein molecules of varying lengths (varying number of amino acids). We extract from the known structures in the PDB chains/fragments of varying lengths.

Our findings are revealing. They show, for instance, that a baseline model, to which we refer as **Vanilla GAN** presents challenges with regards to reaching convergence and, moreover, only captures local patterns/structure in the distance matrices that it generates. We investigate a thorough list of variants of this baseline model in order to improve its convergence and present models that indeed converge better. However, the improvements in convergence do not relate to these models capturing more structure in their generated datasets. Finally, we investigate a GAN variant that makes use of the Wasserstein distance in its loss function. The model, to which we refer as **WGAN**, manages to capture the long-range/distal structure that is the hallmark of realistic protein tertiary structures, but the model has a harder time capturing the short-range structure. As expected, the training datasets of larger distance matrices present more challenges to the baseline model and some of the variants, but not to WGAN. Our evaluation relates many additional observations of use to researchers considering GANs for structure-related studies in computational structural biology. For instance, we show that a convergence-based analysis is necessary but not sufficient to indicate on its own the expected quality of highly-structured data, such as protein tertiary structures.

We make data and selected trained models publicly available through IEEE Dataport (ieee-dataport.org) under DOI 10.21227/m8sa-cz14. The link https://dx.doi.org/10.21227/m8sa-cz14 (accessed on 18 December 2020) provides access to the input/training data, the four top GAN models (further details in Section 3), and the data generated from these models.

We now proceed to relate methodological details in Section 2. The evaluation and obtained results are described in Section 3. The paper concludes in Section 4 with a summary and an exposition of further directions of research.

## 2. Methods

In the interest of brevity, we assume some basic familiarity with neural networks. We first summarize GANs and their training challenges before relating various mechanisms to improve training convergence, resulting in various models we investigate in this study. We then relate the specific setting in which we train and evaluate the resulting models, the data, representation choice, and performance metrics employed.

### 2.1. A Summary of GANs and Their Training

#### 2.1.1. GANs

**Figure 1:**
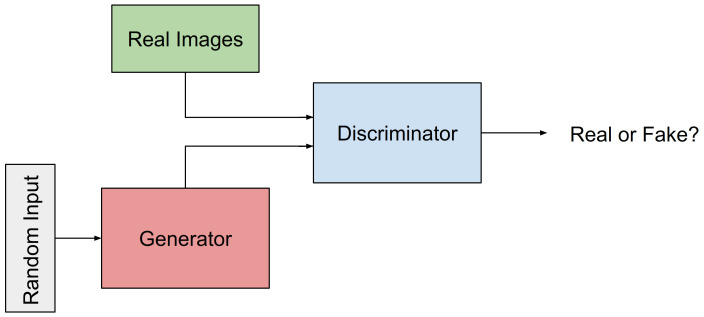
Schematic of the GAN framework, where the Discriminator (**D**) and the Generator (**G**) are trained simultaneously in an adversarial setting. **G** seeks to fool **D** into not being able to distinguish between samples generated from **G** and samples from the training dataset.

GANs were first proposed in [23], where they were first referred to as a framework for generative models trained via an adversarial process. The framework contains two models that are trained simultaneously, a Generative model/Generator, to which we refer as **G** from now on, and a Discriminative model/Discriminator, to which we refer as **D** from now on. The objective for **G** is to capture the data distribution; the objective for **D** is to estimates the probability that a sample comes from the training data rather than **G**. Figure 1 shows the schema of the GAN framework. During training, **G** seeks to maximize the probability of **D** to make a mistake. In contrast, as in a minimax two-player game, **D** seeks to distinguish whether a sample come from the training dataset or from **G**. Work in [23] shows that in the space of arbitrary functions **G** and **D**, a unique solution exists, where **G** recovers the training data distribution, and **D** equals 1/2 everywhere. Goodfellow and co-authors also showed that when **G** and **D** are defined by multilayer perceptrons, the entire GAN system could be trained with backpropagation.

#### 2.1.2. GAN Training

GAN training is a minimax game with the following objective function:

$\min_{G}\;\max_{D}\;V\left(D,G\right)\;=\;E_{x∼p_{\mathrm{data}}\left(x\right)}\left[log\left(D_{x}\right)\right]+E_{z∼p_{z}\left(z\right)}\left[log\left(1-D\left(G\left(x\right)\right)\right)\right]$.

The discriminator **D** tries to maximize the above function, whereas the generator **G** tries to minimize it. In particular, D(x) is **D**’s estimate of the probability that a real data instance *x* is real. $E_{x∼p_{\mathrm{data}}\left(x\right)}$ is the expected value over all the real data instances *x*. G(z) is **G**’s output when given noise *z*. D(G(z)) is **D**’s estimate of the probability that a fake instance is real. $E_{z∼p_{z}\left(z\right)}$ is the expected value over all random inputs *z* to **G**; that is, the expected value over all generated fake instances G(z).

Though in principle training a GAN is a straightforward process, many practical challenges have been identified that to some extent hamper their adoption across application domains. These challenges include non-convergence (where the parameters oscillate, destabilize, and never converge), mode collapse (where **G** collapses and so produces limited varieties of samples), and diminished gradient (where **D** gets too successful too fast, causing **G**’s gradient to vanish and so learn nothing). To address these issues, various training mechanisms have been proposed [21]. In this paper, we consider three reportedly effective ones, such as spectral normalization, virtual batch normalization, and the two-time update rule. Combination of these strategies results in various models that we investigate and evaluate in this study.

### 2.2. Vanilla GAN

**Figure 2:**
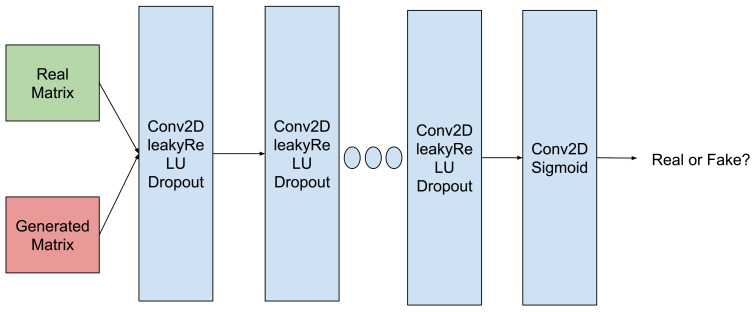
Schematic of **D** employed in this paper, modeled after work in [14,15].

The basic model we investigate for its ability to generate realistic tertiary protein structures is the one proposed recently in [14,15]. We refer to this model as **Vanilla GAN**. **D** in **Vanilla GAN** combines several Conv2D, LeakyReLU, and Dropout layers together to discriminate against training/input and generated data, as shown in Figure 2. As we detail later in this section, our data are distance matrices.

### 2.3. Vanilla GAN Variants to Address Convergence

As summarized above, we consider three main mechanisms via which one aims to improve convergence in **Vanilla GAN**: two-time update rule, spectral normalization, and virtual batch normalization.

#### 2.3.1. Two-Time Update Rule (TTUR)

First proposed in [24] as a mean to achieve the Nash equilibrium, the two-time update rule can be applied to a GAN simply by choosing two different learning rates for **D** and **G**. In [24], the authors achieve a state-of-the-art result using the two-time update rule in popular networks. Typically, having a higher learning rate for **D** ensures that **G** takes smaller steps to learn the distribution and so does not rush to achieve an unrealistic solution in the adversarial setting.

#### 2.3.2. Spectral Normalization (SpecNorm)

First introduced in [25], spectral normalization seeks to address the exploding gradient and mode collapse problem. It does so by controlling the Lipschitz constant (maximum absolute value of the derivatives) of **D**. To be exact, the strategy normalizes the weight *W* for each layer with the spectral norm σ(W), resulting in one being the Lipschitz constant for each layer, as well as the whole network, and bounding of the gradients. Spectral normalization is computationally expedient. One of its most useful applications lies in the efficient computation of the Wasserstein distance in Wasserstein GANs.

#### 2.3.3. Virtual Batch Normalization (VBN)

Virtual Batch Normalization was first presented as one of the techniques to improve GAN training in deep convolutional GANs [21]. A drawback of regular batch normalization is that it causes the output of a neural network to be highly dependent on other inputs of the same minibatch as the original input. To avoid this, the reference batch is chosen at the start of the training and remains fixed. Virtual batch normalization is computationally expensive. For this reason, it is typically applied only on **G**. In this work, we consider three settings, applying it to **D** alone, **G** alone, or applying it to both.

#### 2.3.4. Resulting Vanilla GAN Variants

Considering the three mechanisms above, we construct the following 9 additional models: **Vanilla GAN + TTUR**, **Vanilla GAN + SpecNorm**, **Vanilla GAN + VBN**, **Vanilla GAN + TTUR + SpecNorm**, **Vanilla GAN + TTUR + VBN**, **Vanilla GAN + SpecNorm + VBN**, and **Vanilla GAN + TTUR + SpecNorm + VBN**. This is a thorough list of models that considers one mechanism (3 choose 1), two mechanisms jointly (3 choose 2), and three mechanisms jointly (3 choose 3). With **Vanilla GAN** serving as our baseline, we have a total of 8 models so far.

It is worth noting that the VBN and SpecNorm can in principle be applied to either **D**, **G**, or both. We have experimented with these options, and our findings are in agreement with related literature [21] that VBN is an expensive mechanism, and Spec norm is a method that can be effective to stabilize **D** but ineffective on **G**. Therefore, we limit VBN to **G** only, and SpecNorm to **D** only.

In addition to the 10 GAN models list above, we investigate Wasserstein GAN (**WGAN**), a different GAN that changes the way the loss function is computed and replaces **D** with a critic. We summarize this model below.

### 2.4. Wasserstein GAN: A Promising Model for Tertiary Structures

Wasserstein GAN (WGAN) [26] has been recently proposed in machine learning to improve stability when training GANs. **WGAN** can be considered an extension of the baseline GAN in that it seeks an alternate way of training **G**. Instead of using a Discriminator to classify that a generated sample is real or fake, **WGAN** replaces **D** with a critic; the critic scores the authenticity of a generated sample. This introduction of the critic is motivated by a theoretical argument in [26] that **G** should seek to minimize the distance between the distribution of the training data and the distribution of the generated data. The added benefit of **WGAN** is that the training process is also more stable and less sensitive to the model architecture and choice of hyperparameter configurations. **WGAN** implements three major steps to improve stability. (1) **WGAN** replaces the loss function with the Wasserstein distance, whose gradient is smoother; (2) **WGAN** removes the sigmoid activation function and uses linear activation; (3) **WGAN** clips the gradient to restrict the maximum weight value to be in some range [−c,c], where *c* is a hyperparameter. These changes result in improved stability, solving the possible mode collapse issue, and enable **G** to learn even when the critic performs very well.

### 2.5. Experimental Setup and Architecture

As in [14,15], we employ a distance matrix representation of a tertiary structure. Work in [13,14,15] reports that, when employing angles, the generated angles are not realistic and lead to tertiary structures that are highly distended or in severe self-collisions. A distance matrix encapsulates a tertiary structure as follows: Let us suppose that we are considering chains of length *n* amino acids. As in [14,15], while each amino acid contains many atoms, we simplify these chains by stripping them down to the main-chain carbon (CA) atom contained in each amino acid. So, a tertiary structure of *n* amino acids can be considered as a point in 3n-dimensional space. The corresponding distance matrix can be calculated easily. A matrix of *n* rows and *n* columns stores distances $d_{ij}$ in its entries that specify the Euclidean distance between the CA atom of amino acid *i* and the CA atom of amino acid *j*.

#### Training Dataset(s)

The known protein structures in the PDB contain proteins of varying lengths. So, we consider five settings and thus construct 5 different training datasets. All distance matrices in a given training dataset have the same k×k size, where k∈{6,9,16,64,128}. 115,850 tertiary structures are extracted from the the PDB from the entries listed in [15]. In addition, as in [15], non-overlapping fragments of a given length *l* are sampled from chain ’A’ for each protein structure starting at the first residue. The corresponding distance matrix is calculated and added to the training dataset, which is noted as FL*k*. This process is followed to obtain 115,850 distance matrices in FL6, FL9, and FL16, and 98,966 distance matrices in FL64 and FL128.

### 2.6. Evaluating Generated Datasets

It is important to assess whether the model has learned the reference/training distribution. We do so in various ways.

#### 2.6.1. Evaluating the Presence of Backbone Structure

First, we evaluate the presence of a “backbone” in a generated distance matrix. The backbone places consecutive (i,i+1) CA pairs (of amino acids *i* and i+1 in the sequence) at an ideal distance of 3.83 Å of each-other. There is typically some variation in experimentally-obtained structures around this value, so we round this distance to 4 Å. Using this information, we associate two scores to summarize the “presence of a backbone” in a distance matrix. First, we compute the average Euclidean distance between two consecutive (i,i+1) CA pairs. This corresponds to summing the entries along the main diagonal in a distance matrix and dividing them by the number of entries along the diagonal. We refer to this value as *Average Peptide Bond Length*. Second, we associate with each distance matrix a *Backbone Length Score*, which tallies the number of consecutive CAs that are within 4 Å of each-other. We expect this score to be k−1 for a distance matrix of size *k*.

In each case, the distribution of a particular metric of interest is calculated over the generated dataset, and the distribution is related in terms of summary statistics, such as the mean, median, minimum, and maximum. It is worth noting that the “prediction” of a backbone is a trivial task, in some sense. The order of amino acids in a given protein sequence tells us where the backbone is. In that sense, we do not really need a GAN to learn this structure, as we can recover it from fundamental knowledge of protein architecture. However, it is revealing to understand whether this feature, which is shared across all distance matrices in a training dataset (all experimentally-obtained tertiary structures), is learned by a GAN in the generated dataset or not.

#### 2.6.2. Evaluating the Presence of Local and Distal Structure

The additional off-backbone structure that characterizes tertiary protein structures can be partitioned into two categories, local/short-range versus distal/long-range structure. Each can be quantified with short-range and long-range contacts, respectively. Short-range contacts refer to pairs of amino acids (represented here by their main-chain CA atoms) that are no more than 4 positions away from each-other along the sequence/backbone and are in contact. Long-range contacts refers to pairs of amino acids further away in the sequence/backbone that are in contact. Two amino acids (represented by their main-chain CA atoms) are considered to be in contact if they are spatially proximate. Proximity requires a distance threshold. There are three popular thresholds in literature, 8, 10, and 12 Å. We choose 10 Å for the analysis here. When tallying up the number of short-range contacts as a way of summarizing a distance matrix, the considered amino-acid pairs are (i,i+t), where 1<t≤4 (the lower bound excludes the backbone). When tallying up the number of long-range contacts, the considered amino-acid pairs are (i,i+t), where t>4.

It is worth noting that the prediction/learning of long-range distances/contacts is a more challenging task in protein modeling research. Short-range contacts delineate what is known as the *secondary structure* in protein architecture, which is largely considered to be a solved problem via classical, shallow machine learning models. Nonetheless, it is revealing to understand what well-understood characteristics of realistic tertiary structures a GAN has been able to learn and reproduce in its generated dataset.

The ability to summarize a distance matrix with one score, which gauges the presence of local or distal structure, allows us to then capture a set of distance matrices with a distribution. This in turn permits not only visualization of a training versus a generated dataset but also quantitatively comparison of the (dis)similarity between these datasets as a way of relating the performance of a particular GAN model.

#### 2.6.3. Comparison of Distributions

We make use of several metrics to compare two given distributions, such as the Maximum Mean Discrepancy (MMD), the Bhattacharya distance (BD), and the Earthmover Distance (EMD), which we briefly summarize below.

##### Maximum Mean Discrepancy (MMD)

The MMD test statistic allows measuring the distance between two distributions p(x) and q(y). Briefly, MMD is the largest difference in expectations $\mu_{x}$ and $\mu_{y}$ over functions in the unit ball of a reproducing kernel Hilbert space (RKHS) and is defined as the squared distance between the embeddings in an RKHS H; that is, $\mathrm{MMD}\left(p,q\right)\;=\;|\mu_{x}-\mu_{y}{|}_{H}^{2}$ [27]. MMD has been recently used in training generative adversarial models [28,29] to measure the distance of generated samples to some reference target set. Here, we follow work in [30] to use MMD for model selection, so we can distinguish between different VAE models. Specifically, rather than train a model using the MMD distance to a reference distribution (as opposed to KL divergence, for instance), we use MMD to evaluate the relative performance of various VAE models and find models that generate samples significantly closer to the reference/training distribution.

##### Bhattacharya Distance (BD)

BD [31] measures the distance between two distributions p(x) and q(x) defined over the same domain *X*. It is defined as BD(p,q)=−ln(BC(p,q)). The Bhattcharaya coefficient BC(p,q)=$\sum_{x\in X}\sqrt{p(x)q(x)}$. BC varies from 0 to 1. BD varies from 0 to *∞*.

##### Earth Mover’s Distance (EMD)

EMD [32] is also known as the Wasserstein metric. EMD measures the distance between two probability distributions over a domain. If the distributions are interpreted as two different ways of piling up a certain amount of dirt over the domain, EMD returns the minimum cost of turning one pile into the other. The cost is assumed to be the amount of dirt moved times the distance by which it is moved. EMD can be computed by solving an instance of the transportation problem, using any algorithm for minimum cost flow problem, such as the network simplex algorithm [32].

### 2.7. Implementation Details

Experiments were conducted using the Pytorch framework on 2 Tesla V100 GPU’s in parallel. For **Vanilla GAN**, the learning rate is set to $10^{-4}$ for both **D** and **G**, as in [14]. Binary cross entropy is used as the loss function, and the Adam optimizer is used with beta values of (0.5,0.999). Training times over one training epoch vary from 15.5 s to 146 s. Specifically, considering the FL = 6 and FL = 128 as providing the range and training over one epoch results in 15.5–139.0 s for **Vanilla GAN**, 19.5–146.9 s for **Vanilla GAN + SpecNorm**, 17.8–146.4 s for **Vanilla GAN + VBN**, and 18.7–92.0 s for **WGAN**.

## 3. Results

### 3.1. Experimental Setup

The experimental evaluation is organized in three main parts. First, we analyze the ability of a model to reduce the loss of **D** and **G** and possibly achieve convergence of the two over training epochs. This analysis highlights a few better models and an optimal number of epochs, which are then carried forward and evaluated further in the rest of the analysis. The second part of our analysis then compares the performance of the top models. Specifically, we compare the quality of a generated dataset with the training/reference dataset to determine whether the network has learned the underlying characteristics of the training dataset. We relate this qualitatively and quantitatively, on each of the characteristics, the presence of a backbone, the short-range contacts, and the long-range contacts. The third part of our analysis then relates some actual distance matrices visually for the top models in comparison with experimental data.

### 3.2. PartI: Convergence Analysis

We track the ability of **G** and **D** to reduce loss and converge together over training epochs. The latter vary in {10,20,30,50,70,100}. The Supplementary Material relates this for all 9 models considered, (1) **Vanilla GAN**; (2) **Vanilla GAN + TTUR**; (3) **Vanilla GAN + SpecNorm**; (4) **Vanilla GAN + VBN**; (5) **Vanilla GAN + TTUR + SpecNorm**; (6) **Vanilla GAN + TTUR + VBN**; (7) **Vanilla GAN + SpecNorm + VBN**; (8) **Vanilla GAN + TTUR + SpecNorm + VBN**; and (9) **WGAN**, on each of the five training datasets (FL = 6, FL = 9, FL = 16, FL = 64, and FL = 128).

Here, we relate the performance on the two more challenging datasets, FL = 64 and FL = 128, for **Vanilla GAN**, **Vanilla GAN + SpecNorm**, **Vanilla GAN + VBN**, and **WGAN**.

**Figure 3:**
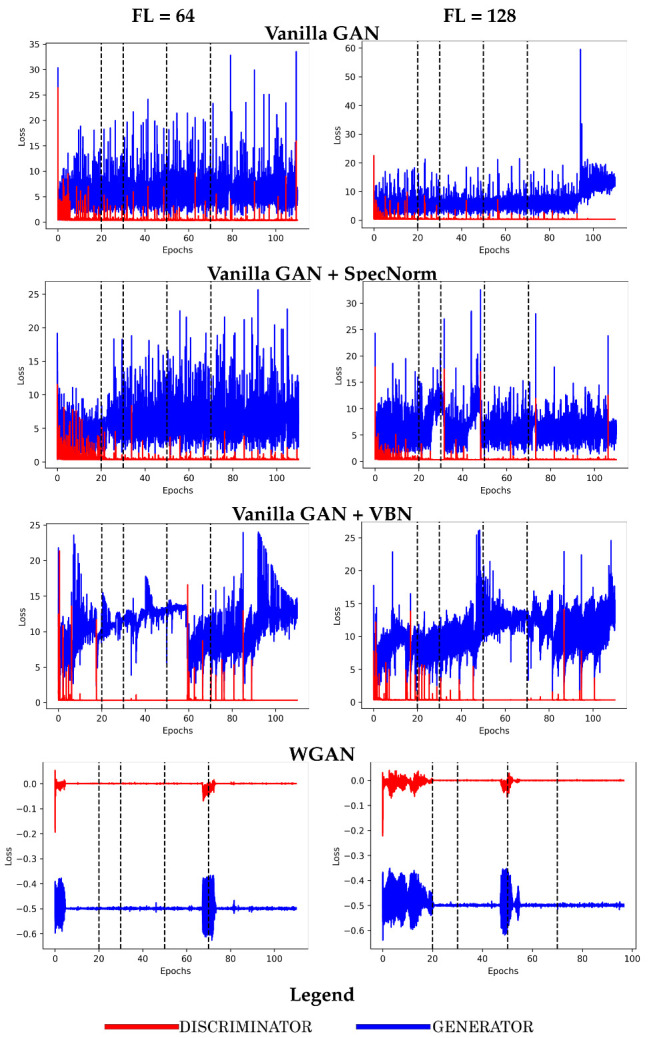
Model performance is related in terms of loss over the FL = 64 and FL = 128 training datasets. Vertical black dashed lines indicate epochs 20, 30, 50, and 70 as possible termination points.

Our detailed analysis on all 9 models on each of the five training datasets indicates that the four models related in Figure 3, **Vanilla GAN**, **Vanilla GAN + SpecNorm**, **Vanilla GAN + VBN**, and **WGAN**, are more consistent in their ability to lower loss in both **D** and **G** across the datasets and have both **D** and **G** be close and even converge to each-other in terms of loss. We note that in the case of **WGAN**, it is possible to obtain negative values for loss; in **WGAN**, the loss function only aims to separate between the scores for real versus synthetic data as larger and smaller.

Analysis of loss also reveals that 50 training epochs are sufficient. The rest of the analysis then focuses on the four models arrested at 50 training epochs and evaluates the quality of their generated datasets in comparison with the training datasets on which they are trained.

### 3.3. Part II: Comparison of the Quality of the Generated Datasets

As summarized above, we first aim to understand the impact of several decisions on the quality of the generated dataset. One of these has to do with the neural network architecture we select. So, we evaluate the top four models listed above on three characteristics: their ability to learn the presence of a backbone, their ability to reproduce short-range structure, and their ability to reproduce long-range structure.

#### 3.3.1. Evaluation of the Learned Backbone Structure

**Figure 4:**
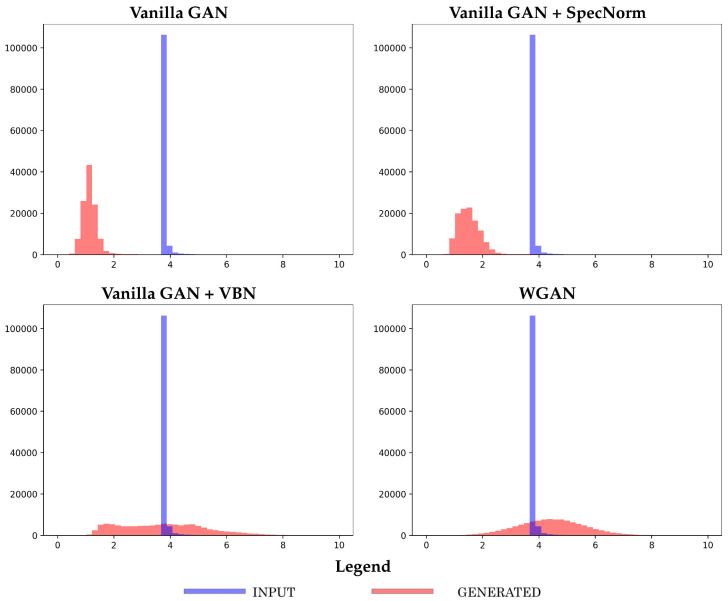
The distribution of the average peptide bond length corresponding to the generated dataset for each of the top four models, **Vanilla GAN**, **Vanilla GAN + SpecNorm**, **Vanilla GAN + VBN**, and **WGAN**, is shown here. The models are trained on the FL = 64 dataset and arrested at 50 training epochs.

First, the average peptide bond length is calculated over a distance matrix, as described in Section 2. The resulting distribution over a training dataset can then be compared to the distribution obtained over a generated dataset. We relate these distributions visually in Figure 4, on a representative dataset, FL = 64, comparing the generated dataset for each of the four models (arrested at 50 training epochs) to the FL = 64 training dataset.

Figure 4 provides valuable information. First, it relates that, as expected, there is variation around the ideal 3.83 Å peptide bond length in the training data, which corresponds to experimentally-known tertiary structures. Second, it shows that **Vanilla GAN** and **Vanilla GAN + SpecNorm** result in tighter distributions but tend to pack consecutive CA atoms closer together than what is experimentally observed. In contrast, there is more variety in the average peptide bond length over the datasets generated by **Vanilla GAN + VBN** and **WGAN**. The distribution obtained by **Vanilla GAN + VBN** seems to be bimodal, whereas that obtained by **WGAN**, while more varied than the ones obtained by **Vanilla GAN** and **Vanilla GAN + SpecNorm**, aligns well with the experimental data. One notices higher peptide bond lengths obtained by **WGAN**, so we proceed with the stricter, complementary evaluation of the presence of a backbone via the backbone length score.

**Figure 5:**
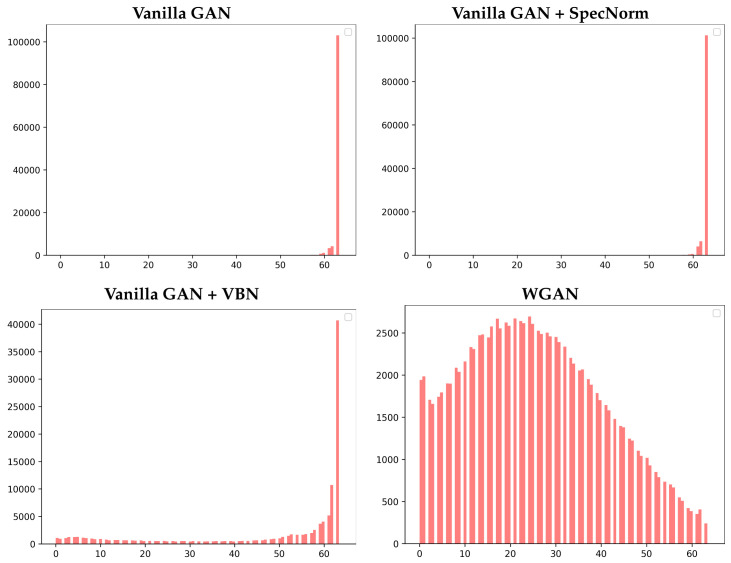
The distribution of the backbone scores corresponding to the generated dataset for each of the top four models, **Vanilla GAN**, **Vanilla GAN + SpecNorm**, **Vanilla GAN + VBN**, and **WGAN**, is shown here. The models are trained on the FL = 64 dataset and arrested at 50 training epochs.

Figure 5 shows the distribution of the backbone length scores corresponding to the generated dataset for each of the four models, **Vanilla GAN**, **Vanilla GAN + SpecNorm**, **Vanilla GAN + VBN**, and **WGAN**. We restrict this visualization-based analysis here to the models trained on the FL = 64 dataset at 50 training epochs.

Figure 5 additionally confirms that **Vanilla GAN** and **Vanilla GAN + SpecNorm** learn the backbone very well; the distributions are narrow and peak at 63 results in a long-tailed distribution. Figure 5 also shows that **WGAN** learns tertiary structures with the correct amount of backbone, but also generates a more diverse distribution consisting of many tertiary structures with non-ideal placements of consecutive CA atoms.

We now summarize the distributions of the backbone length score with summary statistics (mean, median, minimum, and maximum) and do so for all the settings; that is, models arrested at 50 training epochs trained over the FL = 6, FL = 9, FL = 16, FL = 64, and FL = 128 datasets. Table 1 relates these statistics.

Table 1 shows that **Vanilla GAN** and **Vanilla GAN + SpecNorm** learn the backbone well. Both the mean and median are very close to the value FL−1. Some variation is observed on the FL = 64, FL = 16, and FL = 6 datasets, where distance matrices with shorter backbones than expected (see minimum values reported) are present. The performance of both models deteriorates when trained over the datasets of shorter fragments (FL = 6, FL = 9, FL = 16).

Table 1 also shows that **Vanilla GAN + VBN** and **WGAN** underperform in comparison with **Vanilla GAN** and **Vanilla GAN + SpecNorm**. In particular, under this strict metric, **WGAN** captures essentially half or less of the backbone across all datasets. This observation agrees with the distributions related above, which show that there is a broader range in which **WGAN** places consecutive CA atoms in a generated distance matrix.

#### 3.3.2. Evaluation of the Learned Short-Range Structure

**Figure 6:**
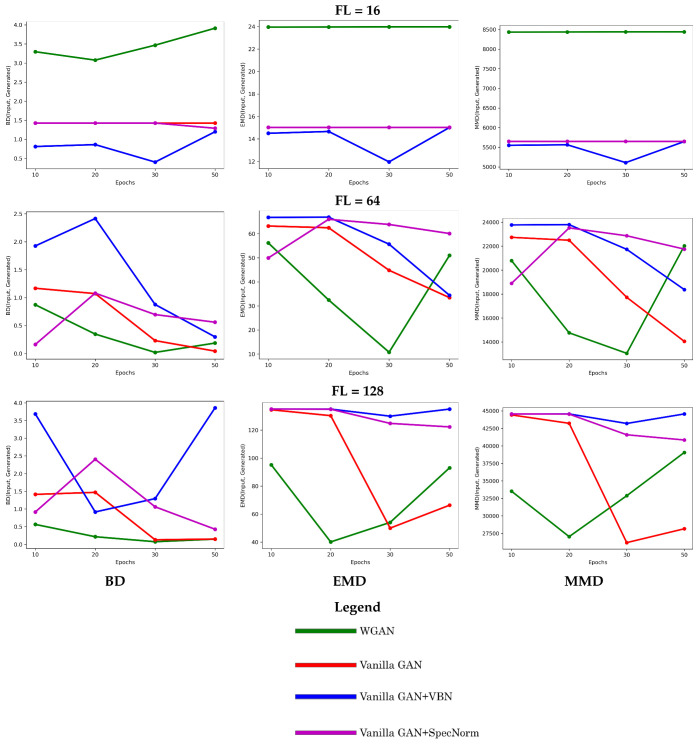
The distribution of the number of short-range contacts in the generated dataset is compared to that in the training dataset via the BD (left panel), EMD (middle panel), and the MMD (right panel) metrics described in Section 2. The progression of these values as a function of the number of training epochs for a specific model is tracked here to show its impact on the quality of the generated dataset. This comparison is conducted separately, for the models trained on the FL = 16, FL = 64, and FL = 128 datasets. Due to the various ranges observed across these metrics and/or across the datasets, the y axes are not scaled.

We additionally summarize the performance of the top four models with respect to the number of short-range contacts. In Figure 6, we compare such distributions over the training versus the generated dataset in terms of BD, EMD, and MMD, and do so for each model over the various training epochs on each of the training datasets. Specifically, the distribution of the number of short-range contacts (computed as in Section 2) is constructed for a training and a generated dataset, respectively, and the two are compared via the distance metrics related in Section 2.

Figure 6 shows that **Vanilla GAN + SpecNorm** and **Vanilla GAN + VBN** perform better in the FL = 16 setting. However, as the size of the distance matrix increases (FL = 64 and FL = 128), **Vanilla GAN** and **WGAN** perform better. In addition, the performance of **Vanilla GAN** improves with further training. Overall, all four models capture short-range contacts well.

**Figure 7:**
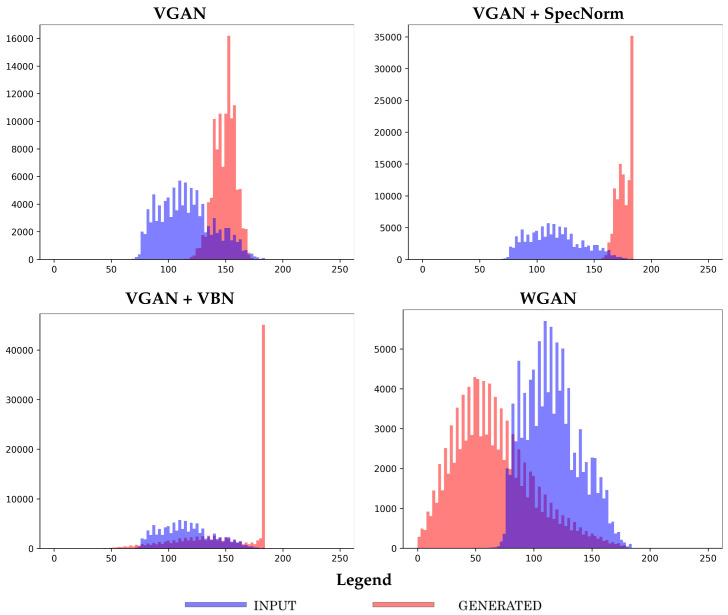
The distribution of the number of short-range contacts corresponding to the generated dataset for each of the top four models, **Vanilla GAN**, **Vanilla GAN + SpecNorm**, **Vanilla GAN + VBN**, and **WGAN**, is shown here. The models are trained on the FL = 64 dataset and arrested at 50 training epochs.

Figure 7 provides more detail and shows the distribution of the short-range contacts corresponding to the generated dataset for each of the top four models, **Vanilla GAN**, **Vanilla GAN + SpecNorm**, **Vanilla GAN + VBN**, and **WGAN**. We restrict this here to the models trained on the FL = 64 dataset. Figure 7 additionally confirms that all four models do well with regards to learning short-range contacts.

#### 3.3.3. Evaluation of the Learned Long-Range/Distal Structure

**Figure 8:**
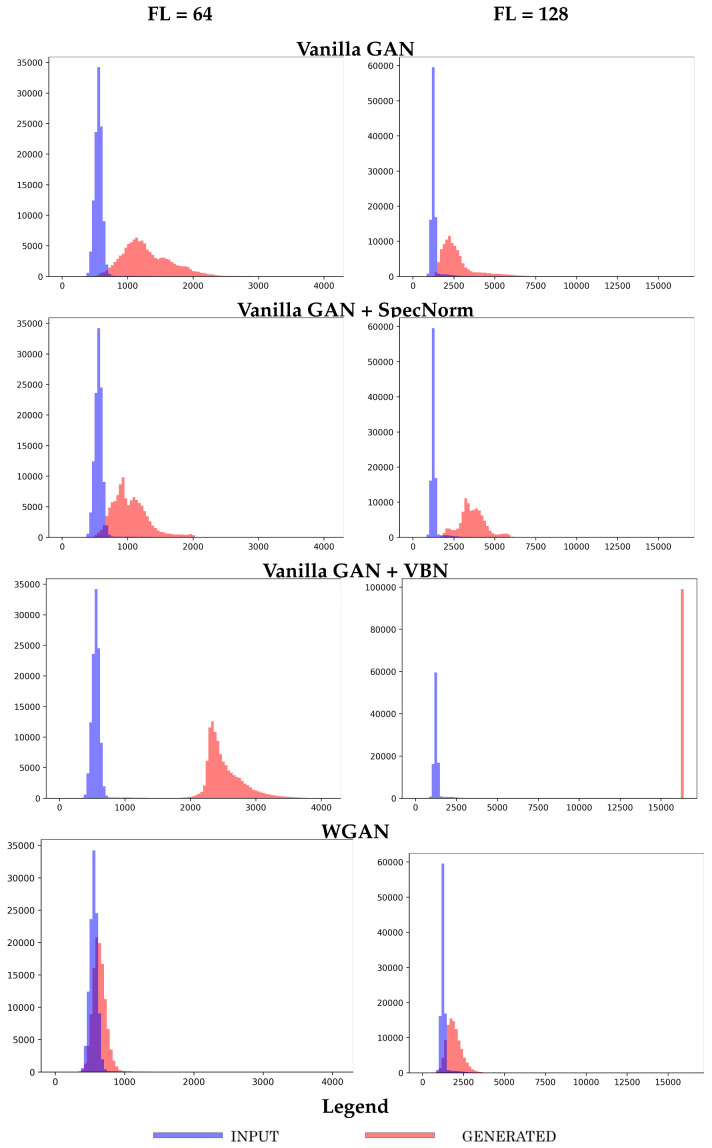
The datasets generated by the top four models are compared to the reference dataset in terms of the number of long-range contacts. The models are trained separately on each of the longer-fragment datasets (FL = 64 and FL = 128) and are arrested here at 50 training epochs.

We first relate a summary observation and compare the four models obtained at 50 training epochs. Figure 8 shows the generated versus the reference histograms of the number of long-range contacts for each of the four models trained, respectively, over the longer-fragment libraries of FL = 64 and FL = 128. Figure 8 shows that the two Vanilla GAN variants underperform in capturing the long-range structure in a distance matrix.

**Figure 9:**
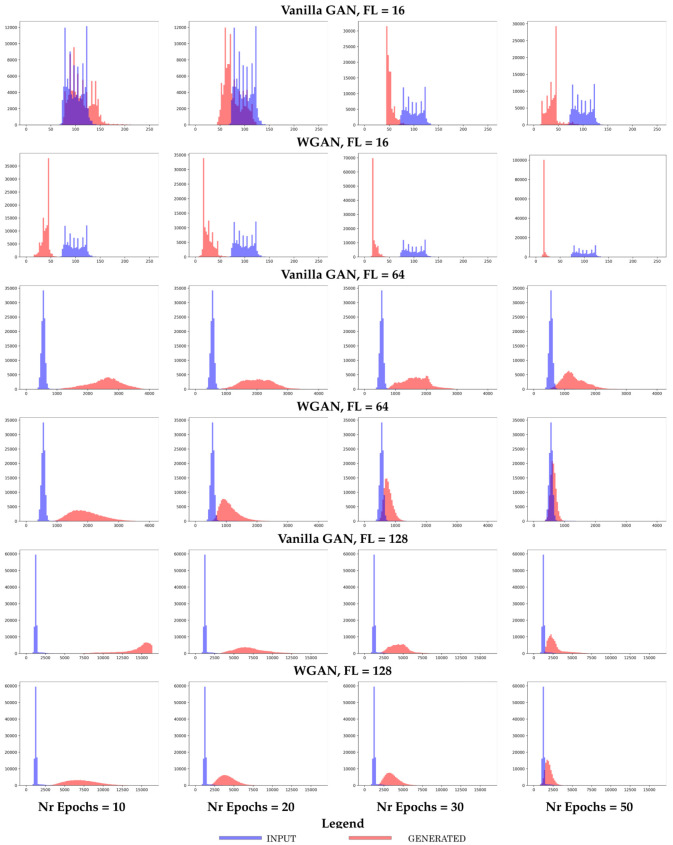
In each plot, the distribution of the number of long-range contacts in the generated dataset is compared to that in the training dataset. This is done for **Vanilla GAN** and **WGAN**, respectively. Results are shown separately, when these models are trained on the FL = 16, FL = 64, and FL = 128 datasets. Results are shown as models are trained, as a function of the number of training epochs, in order to additionally determine an optimal value for the length of the training in terms of its impact on the quality of the generated dataset.

**Vanilla GAN versus WGAN:** The above findings prompt us to investigate more in detail the performance of **Vanilla GAN** and **WGAN**. Paying attention only to convergence in loss can be misleading, as the model can converge in a local optimum of the loss surface. Instead, here we now “arrest” a model at various training epochs varying in {10,20,30,50} and utilize the model learned at each “arrest” point to generate a dataset of the same size (number of fragments) as the training dataset. We show representative results, where we train models separately on the FL = 16, FL = 64, and FL = 128 datasets. As summarized above, for each of these decisions, we compare the generated to the training dataset in terms of the number of long-range contacts, as shown in Figure 9; effectively, each data point/sample is summarized by the number of long-range contacts in it, as described in Section 2.

Several observations can be drawn from Figure 9. First, **Vanilla GAN** performs better than **WGAN** on the shorter fragments (FL = 16 dataset), even though the performance is overall not optimal and worsens with longer training. The negative relationship between the quality of the generated dataset and the longer training is revealing, as it suggests that the model diverges into a sub-optimal part of the loss surface. This divergence is more visible for **WGAN**. In contrast, this behavior is not observed on the longer fragments (FL = 64 and FL = 128). In addition, in these datasets, **WGAN** performs better than **Vanilla GAN**, as it achieves better overlap between the generated and reference datasets.

**Figure 10:**
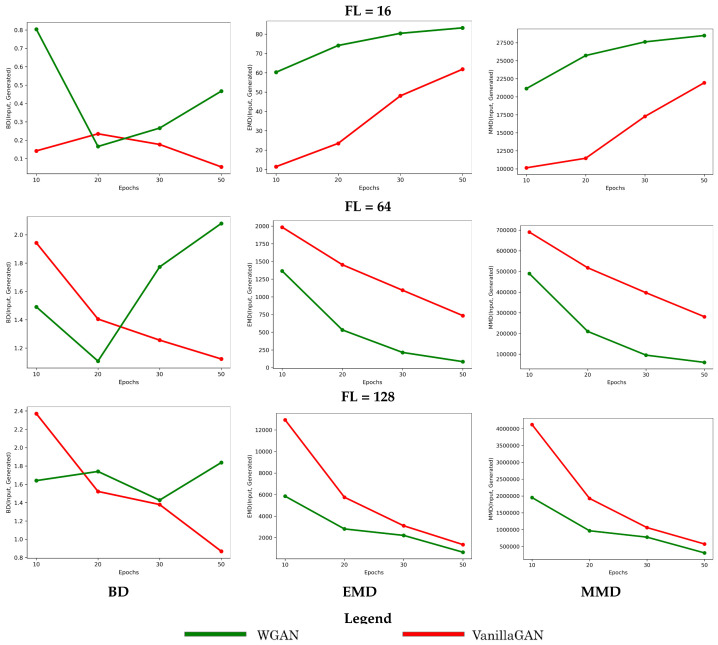
The distribution of the number of long-range contacts in the generated dataset is compared to that in the training dataset via the BD, EMD, and the MMD metrics described in Section 2. The progression of these values as a function of the number of training epochs for a specific model (**Vanilla GAN** versus **WGAN**) is tracked here to show its impact on the quality of the generated dataset. This comparison is conducted separately, for the models trained on the FL = 16, FL = 64, and FL = 128 datasets.

The above observations can be quantified by measuring the distance between the generated and training dataset (the histogram of the number of long-range contacts) via the metrics outlined in Section 2. Figure 10 tracks these distances over the number of training epochs for each model (**Vanilla GAN** versus **WGAN**), trained separately on the FL = 16, FL = 64, and FL = 128 datasets.

Figure 10 allows drawing the same observations from the visual analysis in Figure 9. The divergence of the models (higher in its rate for **WGAN** over **Vanilla GAN**) is obvious with the growing number of epochs on the shorter-length dataset (FL = 16). The improvement of the quality of the generated dataset with longer training is obvious for both models on the longer-fragment datasets (FL = 64 and FL = 128). While the improvement is more drastic (higher rate of change) for **Vanilla GAN**, **WGAN** reaches lower distances overall at each arrest point (over training epochs). Incidentally, this analysis also shows that the results obtained with BD are not as intuitive to interpret; in contrast, EMD and MMD are more robust.

### 3.4. Part III: Visualizing Distance Matrices as Heatmaps

**Figure 11:**
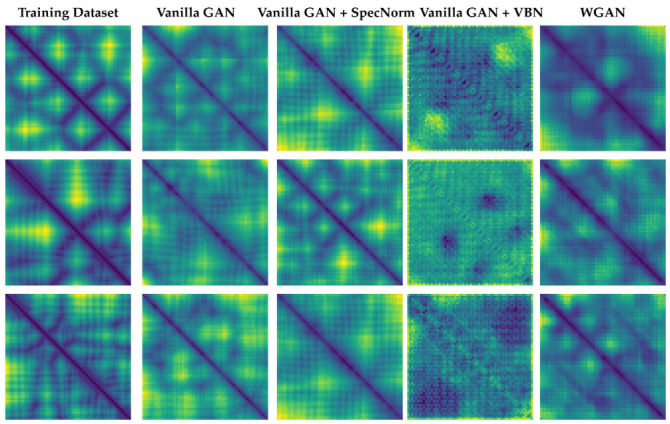
Three sets of distance matrices are selected at random and visualized as heatmaps, with darker colors indicating lower distances. Column 1 shows distance matrices obtained from the training dataset. Columns 2–5 show distance matrices obtained from datasets generated from the various models compared here.

Finally, we show some 64×64 distance matrices generated by each model. These are selected at random over the dataset generated by each model and are shown as heatmaps in Figure 11, with darker colors indicating lower distances.

Figure 11 shows the presence of the backbone in the distance matrices generated by **Vanilla GAN**, **Vanilla GAN + SpecNorm**, and **WGAN**. This is visible as a dark line along the main diagonal. In contrast, the distance matrices obtained by **Vanilla GAN + VBN** lack such structure. Overall, the presence of some additional structure is visible in the distance matrices generated by all the models. Darker regions can be seen off the main diagonal. The distance matrices generated by each of these models, however, vary in quality. In contrast to the other models, **Vanilla GAN + VBN** yields very atypical distance matrices that do not resemble protein-like ones (compare with the left panel). Portions of the backbone are missing, and many off-diagonal regions are dark, indicating that a large number of the CA atoms are proximal. The distance matrices obtained by **WGAN** contain more realistic long-range structure off the diagonal that resembles what is observed in protein tertiary structures (as shown for reference in the leftmost panel).

## 4. Conclusions

The study related in this manuscript seeks to understand the capabilities of foundational GAN architectures that have performed so well in the image domain, such as the baseline **Vanilla GAN** model analyzed here. However, a detailed investigation exposes shortcomings of such architectures. The information present in tertiary structures, even when encoded as distance matrices, is very rich, and the analysis presented here shows that **Vanilla GAN** architectures fail to capture complex patterns, such as long-range contacts, and their performance is highly susceptible to the size of the data objects (to which we refer as fragment length). The study additionally reveals that mechanisms that are touted as effective in stabilizing the training of a GAN model are not all effective, and that performance based on loss alone may be orthogonal to performance based on the quality of generated datasets.

The novel component of the presented study is the demonstration that Wasserstein GAN, to which we refer as **WGAN** in this study, manages to capture both local and distal patterns known as short- and long-range contacts, respectively. The fact that the loss function in WGAN correlates with the quality of the generated distance matrices seems to be key to learning protein-like tertiary structures. However, **WGAN** slightly underperforms in capturing the backbone. To some extent, this is not as problematic, as the location of the backbone is readily given away by the order of the amino acids in the sequence. The more challenging problem is that of learning long-range patterns that cannot be derived from just knowledge of the amino-acid sequence. In this respect, the study in this paper relates a novel finding, that WGAN is effective for generating realistic, protein-like tertiary structures.

We emphasize that the objective of this study is not to seek GANs for the problem of tertiary protein structure prediction, though there is value in exploring alternative approaches to AlphaFold2, particularly models with less inductive bias and leaner models that do not require hundreds of TPUs for training. Though we await an actual publication detailing AlphaFold2, we recognize here the latest development of its superior performance in the latest CASP, which has led many to note that the tertiary protein structure prediction is solved. We note that, while this is a scientific leap, it simplifies the problem of protein structure prediction to the attainment of a single structure.

As we note in Section 1, we have known for a while that proteins are dynamic molecules that switch between different structures under physiological conditions. There has never perhaps been a better time when protein dynamics has been front and center in the public eye; we point here to a growing number of studies reported broadly in media showing the equilibrium motions of the receptor binding domain, the S1 subunit, of the severe acute respiratory syndrome coronavirus 2 (SARS-CoV-2) spike glycoprotein between a closed to a partially-open structure being key to its ability to bind to the human Angiotensin-converting enzyme 2 (ACE2) receptor and so mediate viral entry in human host cells [33,34,35]. Finding the structures that a protein accesses to regulate interactions with molecular partners in the cell is an important problem. Obtaining a broad view of the structure space is thus highly informative. We consider GANs as viable mechanisms to obtain such a view, though the work, as we show in this paper, is in its infancy, and much remains to be done. There is often interest in obtaining a view of the structure space in atomistic detail. While we are eager to investigate end-to-end platforms that can directly generate tertiary structures in the Cartesian representation in future work, in this paper we utilize the distance matrix representation. Given a distance matrix (of distances of pairs of CA atoms), there are multiple approaches to generate corresponding Cartesian Coordinates. For instance, one can utilize the ADMM algorithm, which stands for “Alternating Direction Method of Multipliers” [36]. ADMM combines dual decomposition and the technique of multipliers and is based on a formulation of the tertiary structure reconstruction problem as a convex optimization one [37]. Our experimentation with ADMM (beyond the scope of this paper) reveals that the algorithm often fails to complete, particularly on large distance matrices corresponding to fragments of 64 and longer. In contrast, protocols can be setup in Rosetta that utilize the distances in the distance matrix as restraints for a simulated annealing algorithm seeking Cartesian Coordinates that optimize a restraint-based scoring function. This approach presents its challenges, as well, as it may result in sub-optimal solutions that may not satisfy all the specified restraints.

In principle, the process of adding more resolution (more atoms) is well understood. There are many other options available to researchers to fill in missing backbone atoms given CA atoms. We point, for instance, to BBQ [38], which is one of the top backbone reconstruction protocols. Once the backbone is built, side chains can then packed onto the reconstructed backbone via the Rosetta relax protocol [39] or the SCWRL software, both of which yield low-energy atomistic tertiary structures.

Investigating how redundancy in the input dataset affects the generated dataset is an interesting direction of future work. While the collection of the over 100K tertiary structures we employ to construct the training dataset in this paper is certainly redundant, the training dataset itself is under-sampled in the extraction/sampling of fragments. In contrast, if one constructs a non-redundant dataset (reducing sequence identity and additionally using SCOP or CATH classification as guidance to reduce structure redundancy, as well), then one can better utilize a structure to sample many fragments from it. It is important that the GAN see diverse examples of realistic small pieces.

Many other directions of future research present themselves, such as conditioning generative models on specific amino-acid sequences, relaxing the condition of having fixed-size objects in the training dataset, exploring loss functions that encode multiple objectives related to capturing backbones, local, and distal structure precisely, as well as building end-to-end models that start with tertiary structures and end with tertiary structures.

Data and Model Availability: The reader can find datasets and models at https://dx.doi.org/10.21227/m8sa-cz14 (accessed on 18 December 2020), which is housed at IEEE Dataport. Specifically, the input dataset listing all PDB ids, alongside with code to extract and build the training dataset for a given fragment length (to which we refer as FL throughout this paper), is publicly-available at this link. In addition, the reader can download all the top four models (**Vanilla GAN**, **Vanilla GAN + SpecNorm**, **Vanilla GAN + VBN**, and **WGAN**), arrested/saved at different training epochs; namely, at epoch 10, 20, 30, 50, 70, and 100. Generated data are also available at the same link. We restrict the latter to the data generated from the top two models, **Vanilla GAN** and **WGAN**, arrested at epoch 50.

## Figures and Tables

**Table 1:** For each fragment length (training dataset), the generated distribution of BackboneScores is computed and summarized via its mean, median, minimum, and maximum values.

FL	Vanilla GAN			
	Mean	Med	Min	Max
128	125.31	126	94	127
64	62.82	63	48	63
16	12.75	13	9	15
9	4.02	4	0	6
6	2.93	3	0	4
**FL**	**Vanilla GAN + SpecNorm**			
	**Mean**	**Med**	**Min**	**Max**
128	125.04	126	101	127
64	62.83	63	18	63
16	12.62	13	11	14
9	5.99	6	3	6
6	0.01	0	0	2
**FL**	**Vanilla GAN + VBN**			
	**Mean**	**Med**	**Min**	**Max**
128	127.00	127.0	127	127
64	49.08	61	0	63
16	15.00	15	13	15
9	7.94	8	4	8
6	4.98	5	3	5
**FL**	**WGAN**			
	**Mean**	**Med**	**Min**	**Max**
128	51.94	49	0	127
64	25.56	24	0	63
16	0.005	0	0	10
9	5.52	6	0	8
6	0.47	0	0	5

## Data Availability

Training and generated data, as well as the top trained models, are publicly-available at ieee-dataport.org under DOI 10.21227/m8sa-cz14 and can be downloaded directly at https://dx.doi.org/10.21227/m8sa-cz14 (accessed on 18 December 2020).

## Supplementary Materials

The following are available online, Figure S1: Part I: Performance over the FL = 6, FL = 9, and FL = 16 training datasets, respectively, is shownhere in terms of the average loss over each training dataset. Loss is tracked over training epochs andshown for both the disriminator and generator. Vertical red lines indicate epochs 20, 30, 50, and 70 aspossible termination points; Figure S2: Part II: More models are related here in terms of their loss over the FL = 6, FL = 9, and FL = 16 training datasets. Vertical red lines indicate epochs 20, 30, 50, and 70 as possible termination points; Figure S3: Part III: Performance over the FL = 64 and FL = 128 training datasets, respectively, is shownhere in terms of the average loss over each training dataset. Loss is tracked over training epochs andshown for both the disriminator and generator. Vertical red lines indicate epochs 20, 30, 50, and 70 aspossible termination points; Figure S4: Part IV: More models are related here in terms of their loss over the FL = 64 and FL = 128 training datasets. Vertical red lines indicate epochs 20, 30, 50, and 70 as possible termination points; Figure S5: The performance of WGAN is related here in a similar manner, in terms of loss over eachof the training datasets. Vertical red lines indicate epochs 20, 30, 50, and 70 as possible terminationpoints. Recall that in WGAN, the loss function only aims to separate between the scores for real versussynthetic data as larger and smaller, so negative values can be obtained.

## Sample Availability

Samples of the compounds are not available from the authors.

## Abbreviations

The following abbreviations are used in this manuscript:

ADMM	Alternating Direction Method of Multipliers
BBQ	Backbone Building from Quadrilaterals
BD	Bhattacharya distance
CA	Alpha Carbon
CASP	Critical Assessment of protein Structure Prediction
EMD	Earthmover Distance
GAN	Generative Adversarial Networks
MMD	Maximum Mean Discrepancy
PDB	Protein Data Bank
SpecNorm	Spectral Normalization
TTUR	Two-Time Update Rule
VBN	Virtual Batch Normalization
WGAN	Wasserstein GAN
