# Supplementary material for: Generative Adversarial Learning of Protein Tertiary Structures
Source: Molecules. 2021 Feb 24;26(5):1209. doi: 10.3390/molecules26051209 (PMC7956369; doi:10.3390/molecules26051209)
Supplement: Supplementary file 1 [file molecules-26-01209-s001.pdf]

Article

Supplementary Material
Generative Adversarial Learning of Protein Tertiary Structures

Taseef Rahman[1], Yuanqi Du[1], Liang Zhao[2], and Amarda Shehu[1,3,4,5,*]

1 Department of Computer Science, George Mason University, Fairfax, VA 22030, USA; trahman2@gmu.edu; amarda@gmu.edu
2 Department of Computer Science, Emory University
3 Center for Advancing Human-Machine Partnerships, George Mason University
4 Department of Bioengineering, George Mason University
5 School of Systems Biology, George Mason University
* Correspondence: amarda@gmu.edu
† Current address: 4400 University Dr., MS 4A5, Fairfax, VA 22030, USA.

Version December 18, 2020 submitted to Molecules

Loss-Based Analysis

As related in the main manuscript, we track the ability of the generator and discriminator to reduce loss and converge together over training epochs. We do so for each of the models considered in this study and respectively for each of the training datasets on which the models are trained. Figures 1-5 relate this loss-based analysis. As related in the main manuscript, models where both the generator and the discriminator converge to similar loss values, particularly on datasets with the higher fragment lengths, are **Vanilla GAN**, **Vanilla GAN + VBN**, **Vanilla GAN + SpecNorm**, and **WGAN**. These models are analyzed further in terms of their performance on several metrics of interest in the main manuscript.

© 2020 by the authors. Submitted to *Molecules* for possible open access publication under the terms and conditions of the Creative Commons Attribution (CC BY) license (http://creativecommons.org/licenses/by/4.0/).

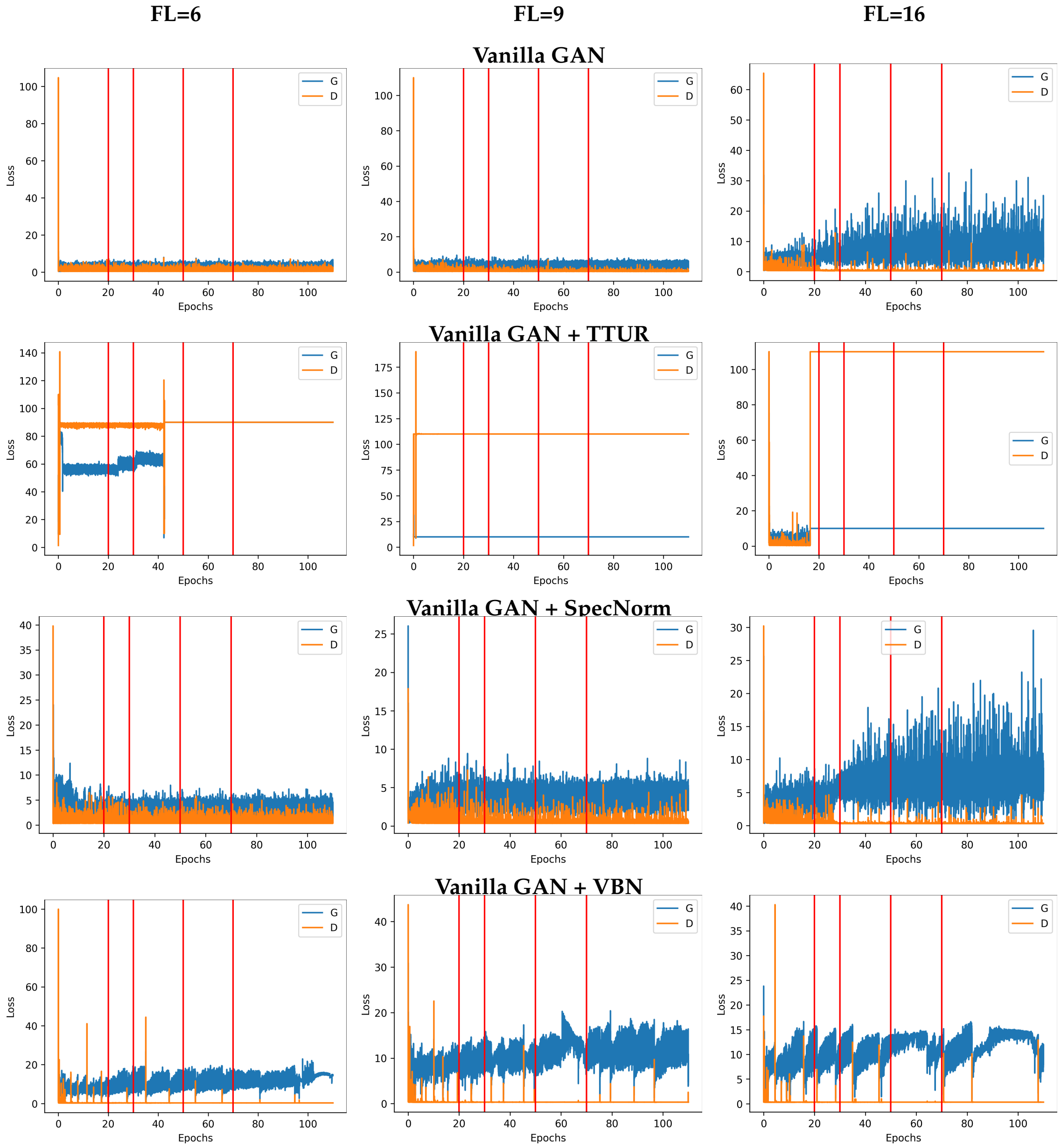

Figure 1. Part I: Performance over the FL=6, FL=9, and FL=16 training datasets, respectively, is shown here in terms of the average loss over each training dataset. Loss is tracked over training epochs and shown for both the disriminator and generator. Vertical red lines indicate epochs 20, 30, 50, and 70 as possible termination points.

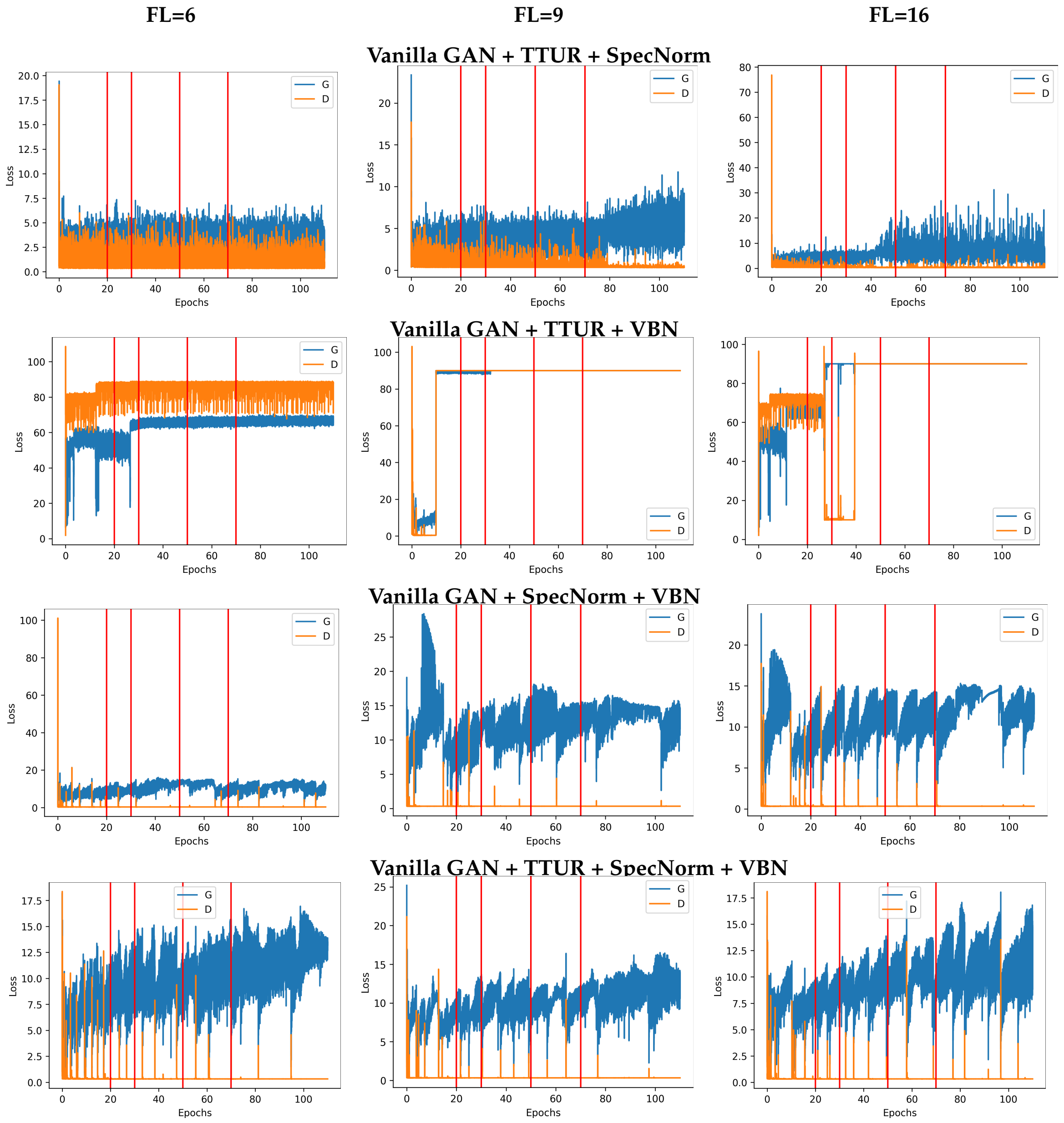

Figure 2. Part II: More models are related here in terms of their loss over the FL=6, FL=9, and FL=16 training datasets. Vertical red lines indicate epochs 20, 30, 50, and 70 as possible termination points.

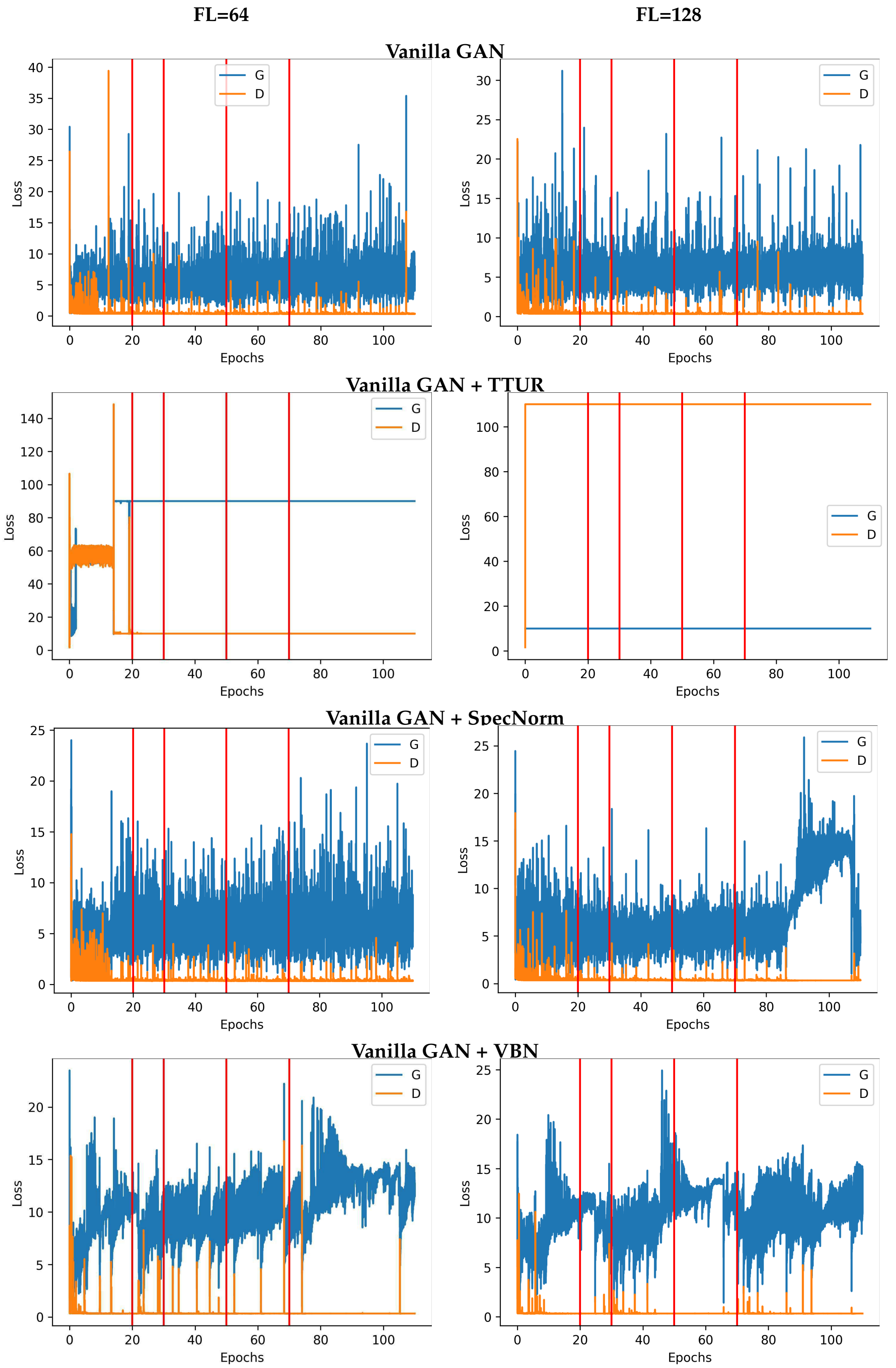

Figure 3. Part III: Performance over the FL=64 and FL=128 training datasets, respectively, is shown here in terms of the average loss over each training dataset. Loss is tracked over training epochs and shown for both the disriminator and generator. Vertical red lines indicate epochs 20, 30, 50, and 70 as possible termination points.

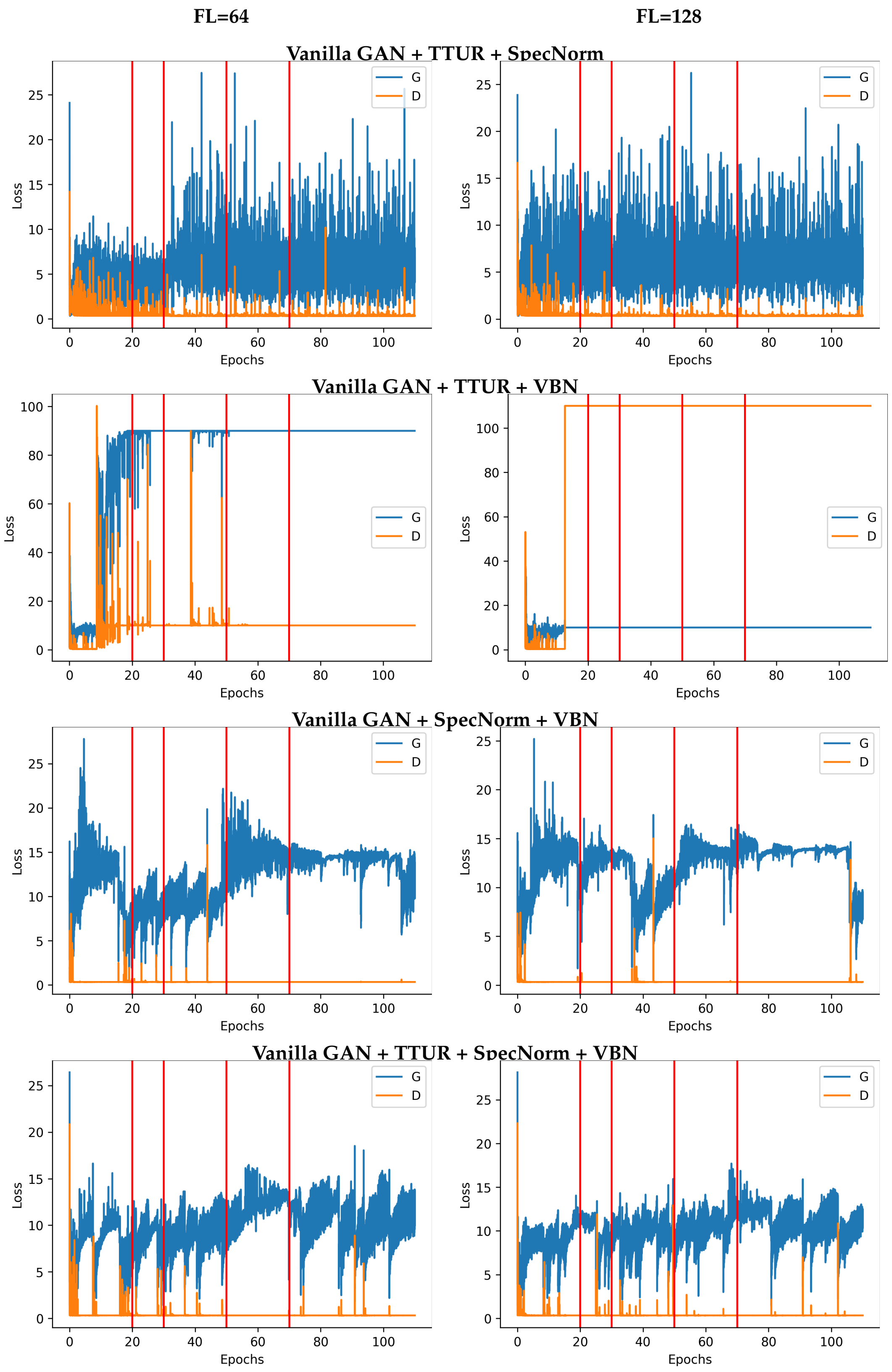

Figure 4. Part IV: More models are related here in terms of their loss over the FL=64 and FL=128 training datasets. Vertical red lines indicate epochs 20, 30, 50, and 70 as possible termination points.

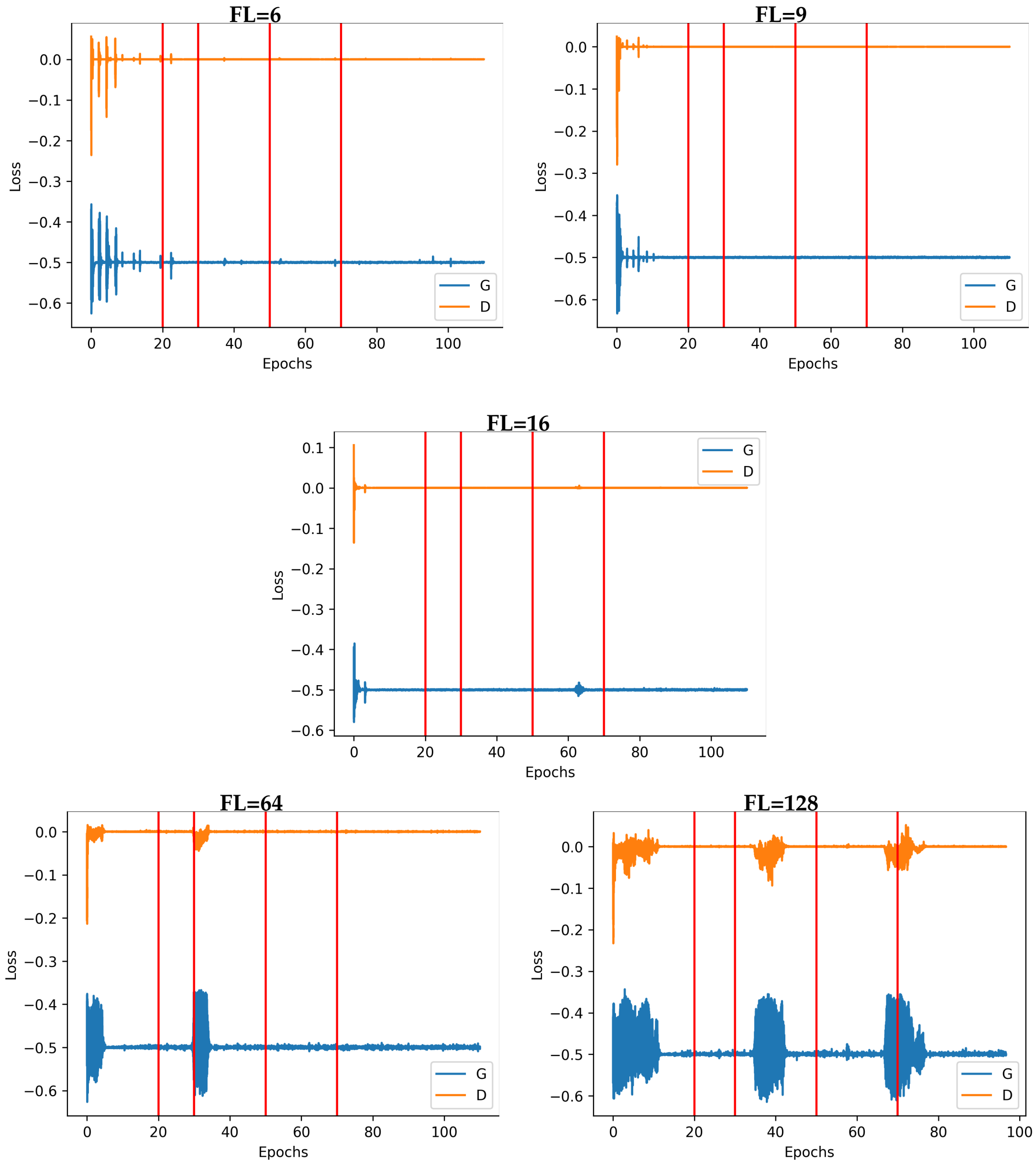

Figure 5. The performance of WGAN is related here in a similar manner, in terms of loss over each of the training datasets. Vertical red lines indicate epochs 20, 30, 50, and 70 as possible termination points. Recall that in WGAN, the loss function only aims to separate between the scores for real versus synthetic data as larger and smaller, so negative values can be obtained.
